# Supplementary material for: Biology of Two-Spotted Spider Mite (Tetranychus urticae): Ultrastructure, Photosynthesis, Guanine Transcriptomics, Carotenoids and Chlorophylls Metabolism, and Decoyinine as a Potential Acaricide
Source: Int J Mol Sci. 2023 Jan 15;24(2):1715. doi: 10.3390/ijms24021715 (PMC9864819; doi:10.3390/ijms24021715)

Supplementary Figure S1: UV-VIS spectra of the chlorophyll and pheophytin in healthy Lima bean leaves, TSSM-fed Lima bean leaves, and TSSM via HPLC-DAD (λ: 665 nm). Numbers refer to: Chlorophyll b (1); Chlorophyll b’ (2); Chlorophyll a (3); Chlorophyll a’ (4); Pheophytin b’ (5); Pheophytin b (6); Pheophytin a’ (7); Pheophytin a (8).

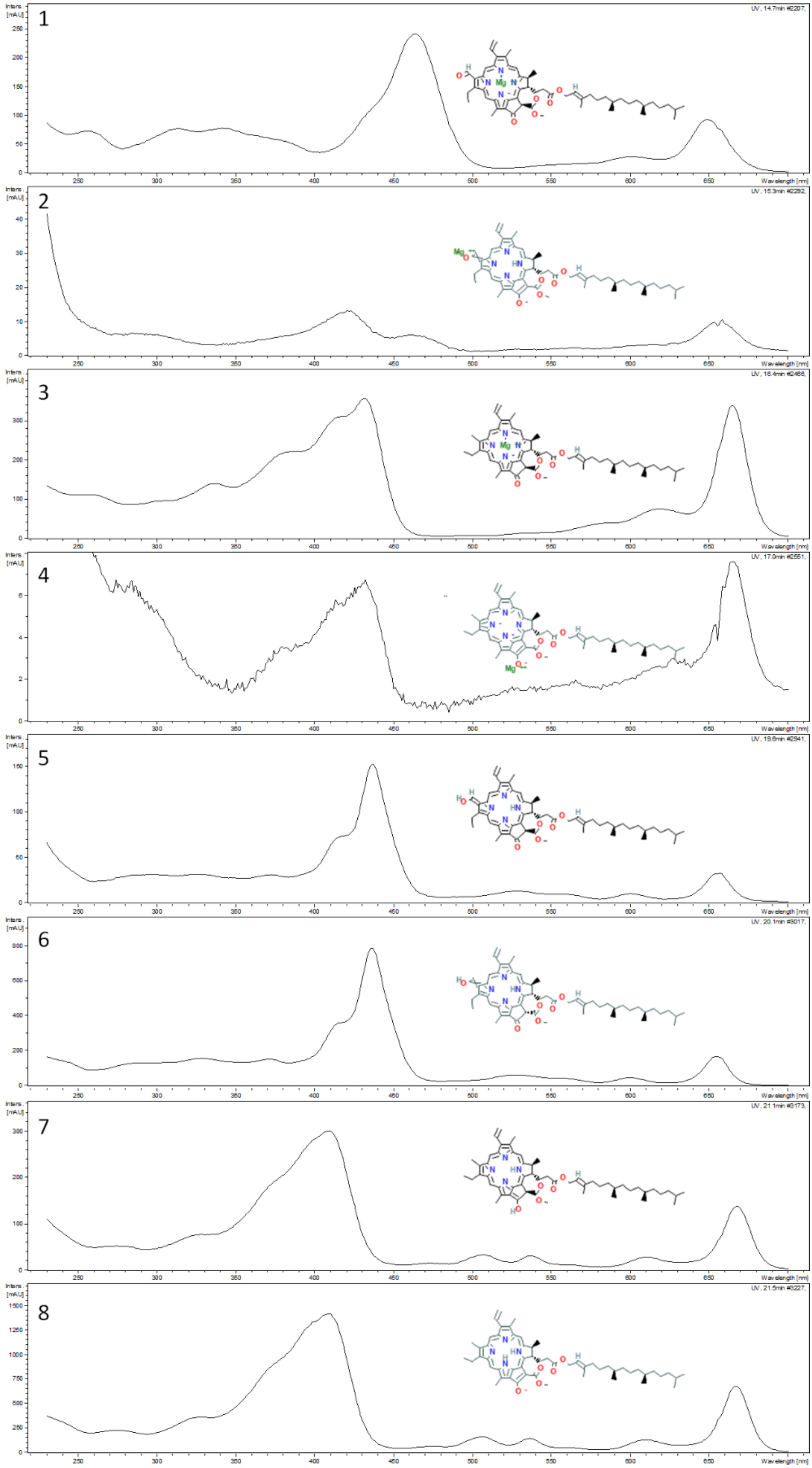

Supplement: Supplementary file 1 [file ijms-24-01715-s001.zip › Supplementary Figure S1.pdf]
