# Supplementary material for: Biology of Two-Spotted Spider Mite (Tetranychus urticae): Ultrastructure, Photosynthesis, Guanine Transcriptomics, Carotenoids and Chlorophylls Metabolism, and Decoyinine as a Potential Acaricide
Source: Int J Mol Sci. 2023 Jan 15;24(2):1715. doi: 10.3390/ijms24021715 (PMC9864819; doi:10.3390/ijms24021715)

**Supplementary Figure S2:** UV-VIS spectra of the identified carotenoids in healthy Lima bean leaves, TSSM-fed Lima bean leaves, and TSSM via HPLC-DAD (λ: 450 nm). Numbers refer to: Lutein (1); unidentified carotenoid#1 (2); 15-cis-β-carotene (3); 13-cis-β-carotene (4); trans-α-carotene (5); trans-β-carotene (6); cis-α-carotene (7); 9-cis-β-carotene (8); γ-carotene (9).

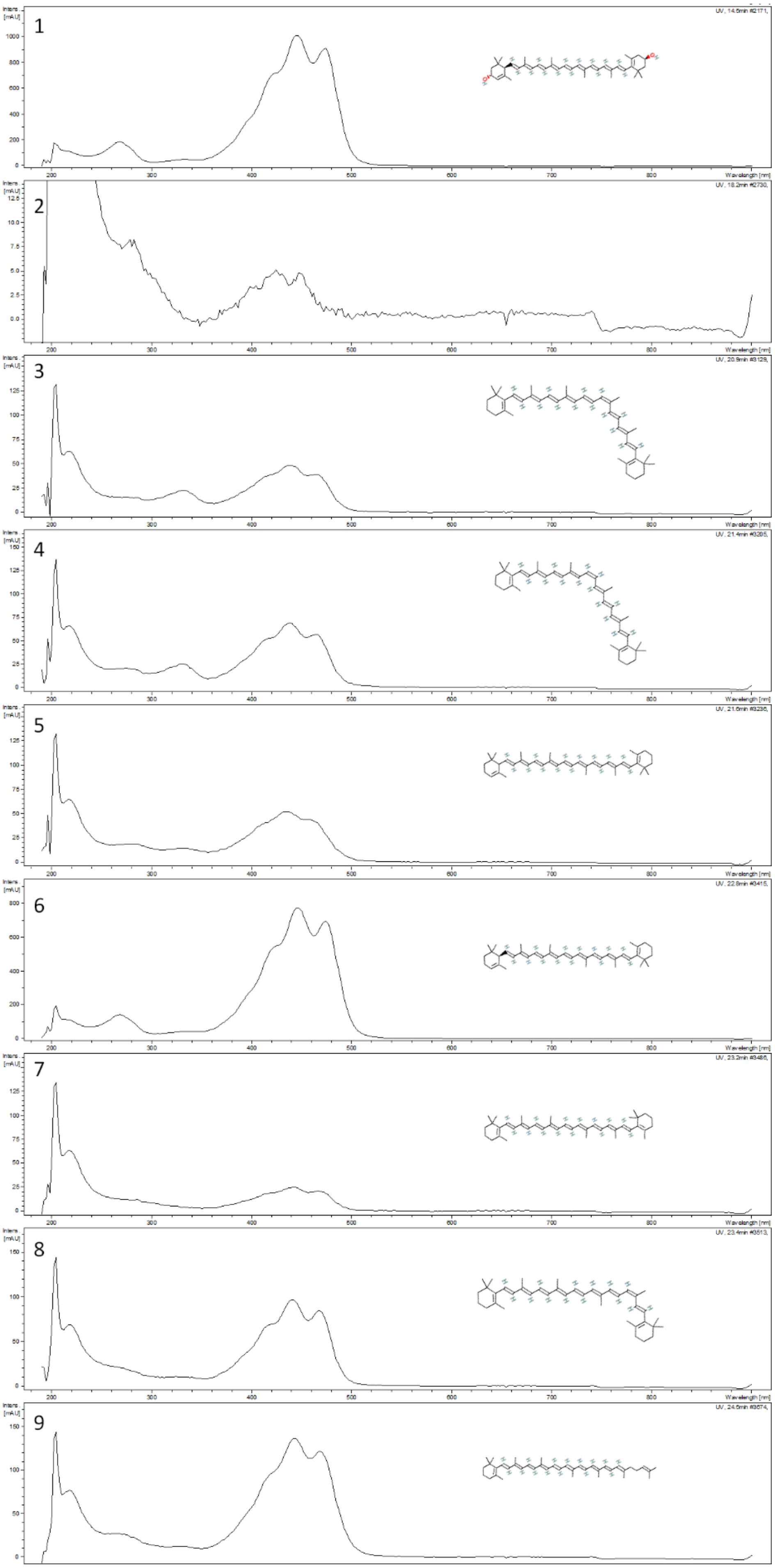

Supplement: Supplementary file 1 [file ijms-24-01715-s001.zip › Supplementary Figure S2.pdf]
