# Supplementary material for: Biology of Two-Spotted Spider Mite (Tetranychus urticae): Ultrastructure, Photosynthesis, Guanine Transcriptomics, Carotenoids and Chlorophylls Metabolism, and Decoyinine as a Potential Acaricide
Source: Int J Mol Sci. 2023 Jan 15;24(2):1715. doi: 10.3390/ijms24021715 (PMC9864819; doi:10.3390/ijms24021715)
Supplement: Supplementary file 1 [file ijms-24-01715-s001.zip › Supplementary Figure S3.pdf]

SUPPLEMENTARY FIGURE S3. BIOANALYZER 2100 expert_Protein 80_DE34903611

Assay Class: Protein 80

Created: 06/12/2022 3:58:51 PM
Modified: 07/12/2022 3:16:48 PM

Electrophoresis File Run Summary

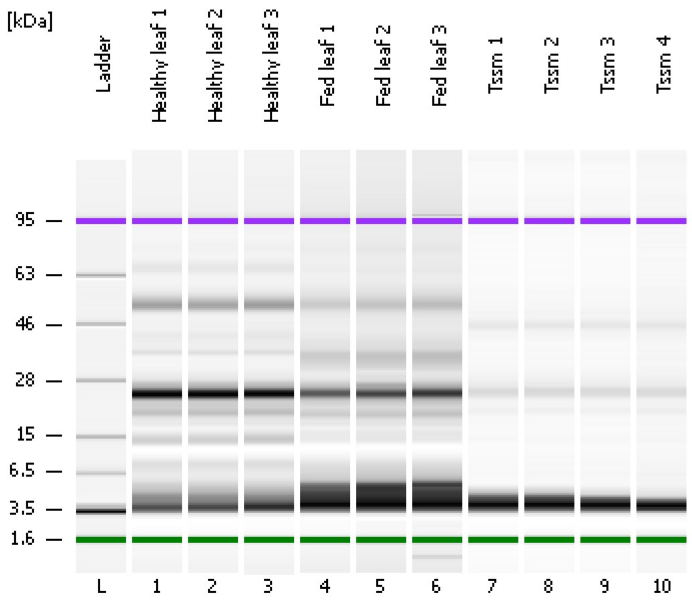

Instrument Information:

Instrument Name: DE34903611

Firmware: C.01.069

Serial#: DE34903611

Type: G2938C

Assay Information:

Assay Origin Path: C:\Program Files\Agilent\2100 bioanalyzer\2100 expert\assays\protein\Protein 80 Series II.xsy

Assay Class: Protein 80

Version: 4.7

Assay Comments: Protein Analysis 5 - 80 kDa

© Copyright 2003 - 2009 Agilent Technologies, Inc.

Chip Information:

Chip Lot #:

Reagent Kit Lot #:

Chip Comments:

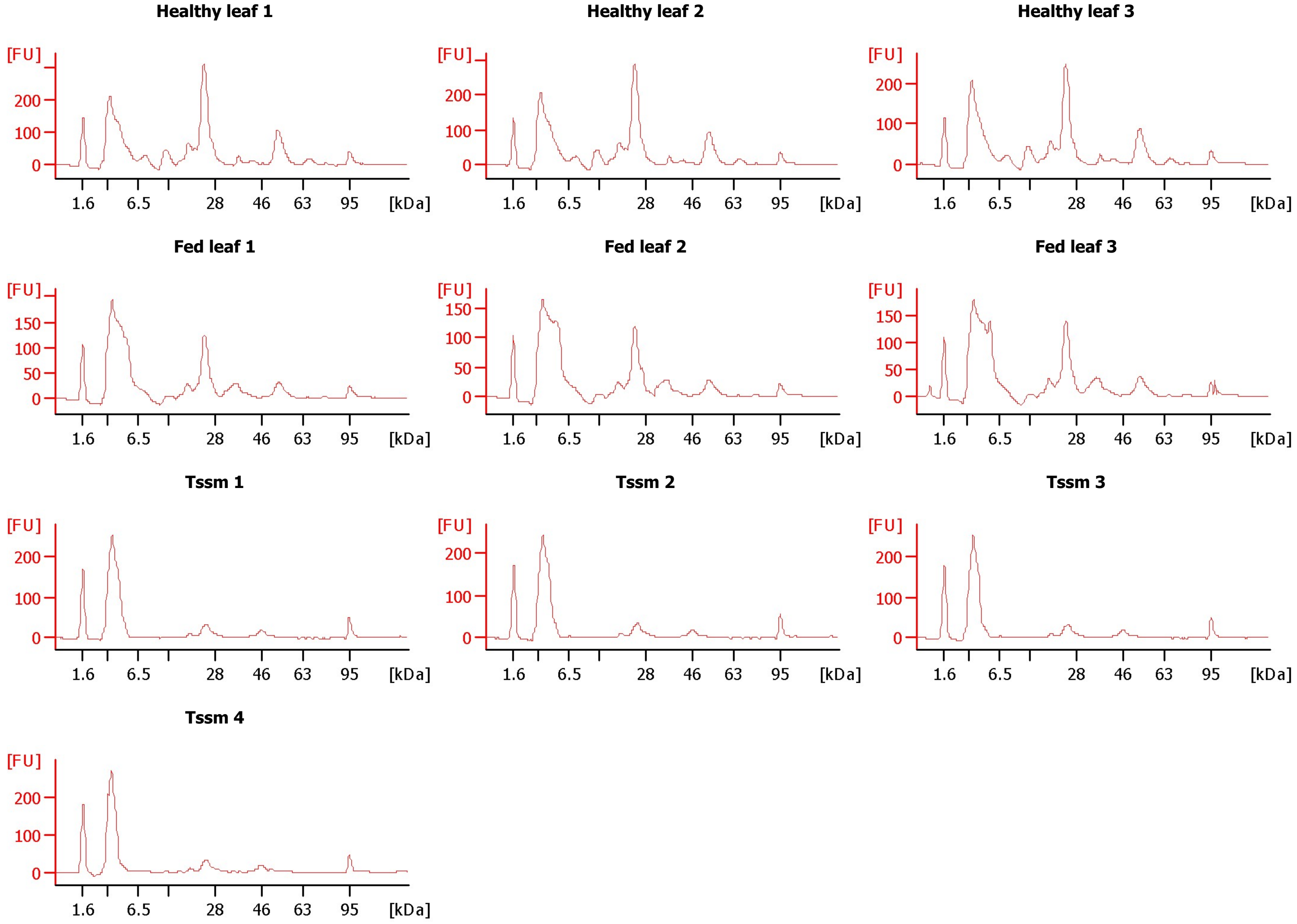

© Copyright 2003 - 2009 Agilent Technologies, Inc.
