# Supplementary material for: Biology of Two-Spotted Spider Mite (Tetranychus urticae): Ultrastructure, Photosynthesis, Guanine Transcriptomics, Carotenoids and Chlorophylls Metabolism, and Decoyinine as a Potential Acaricide
Source: Int J Mol Sci. 2023 Jan 15;24(2):1715. doi: 10.3390/ijms24021715 (PMC9864819; doi:10.3390/ijms24021715)
Supplement: Supplementary file 1 [file ijms-24-01715-s001.zip › Supplementary Table S1.pdf]

Supplementary Table S1: Tukey's HSD post hoc differences in the content of chlorophyll and chlorophyll degradation products. *P<0.05; **P<0.01; ***P<0.001

	Sample(s)		hsd post hoc
chlorophyll a	Healthy Leaf	TSSM-fed leaf	14.859***
		TSSMs	75.889***
	TSSM-fed leaf	TSSMs	61.03***
chlorophyll a'	Healthy Leaf	TSSM-fed leaf	5.396***
		TSSMs	13.222***
	TSSM-fed leaf	TSSMs	7.826***
pheophytin a	Healthy Leaf	TSSM-fed leaf	-0.141*
		TSSMs	-3.58***
	TSSM-fed leaf	TSSMs	-3.439***
pheophytin a'	Healthy Leaf	TSSM-fed leaf	-0.896
		TSSMs	-38.331***
	TSSM-fed leaf	TSSMs	-37.435***
chlorophyll b	Healthy Leaf	TSSM-fed leaf	5.28***
		TSSMs	19.683***
	TSSM-fed leaf	TSSMs	14.403***
chlorophyll b'	Healthy Leaf	TSSM-fed leaf	0.637***
		TSSMs	0.676***
	TSSM-fed leaf	TSSMs	0.039
pheophytin b	Healthy Leaf	TSSM-fed leaf	n.d.
		TSSMs	n.d.
	TSSM-fed leaf	TSSMs	-0.353***
pheophytin b'	Healthy Leaf	TSSM-fed leaf	n.a.
		TSSMs	n.a.
	TSSM-fed leaf	TSSMs	-4.599***
TChC	Healthy Leaf	TSSM-fed leaf	23.478***
		TSSMs	60.54***
	TSSM-fed leaf	TSSMs	37.062***

n.a. "not applicable" due to lack of the compound in the sample
