# Supplementary material for: Biology of Two-Spotted Spider Mite (Tetranychus urticae): Ultrastructure, Photosynthesis, Guanine Transcriptomics, Carotenoids and Chlorophylls Metabolism, and Decoyinine as a Potential Acaricide
Source: Int J Mol Sci. 2023 Jan 15;24(2):1715. doi: 10.3390/ijms24021715 (PMC9864819; doi:10.3390/ijms24021715)
Supplement: Supplementary file 1 [file ijms-24-01715-s001.zip › Supplementary Table S2.pdf]

Supplementary Table S2: Tukey's HSD post hoc differences in the content of carotenoids. *P<0.05; **P<0.01; ***P<0.001

	Sample(s)		hsd post hoc
Lutein	Healthy Leaf	TSSM-fed leaf	-39.391***
		TSSMs	n.a.
	TSSM-fed leaf	TSSMs	n.a.
Putative xanthophyll	Healthy Leaf	TSSM-fed leaf	1.752
		TSSMs	-3.509***
	TSSM-fed leaf	TSSMs	-5.261***
15-*cis*-β-carotene	Healthy Leaf	TSSM-fed leaf	0.714*
		TSSMs	-19.766***
	TSSM-fed leaf	TSSMs	-20.48***
13-*cis*-β-carotene	Healthy Leaf	TSSM-fed leaf	1.975**
		TSSMs	-39.415***
	TSSM-fed leaf	TSSMs	-41.39***
trans-α-carotene	Healthy Leaf	TSSM-fed leaf	4.909***
		TSSMs	-4.356***
	TSSM-fed leaf	TSSMs	-9.265***
cis-α-carotene	Healthy Leaf	TSSM-fed leaf	-6.814***
		TSSMs	-8.531***
	TSSM-fed leaf	TSSMs	-1.717***
trans-β-carotene	Healthy Leaf	TSSM-fed leaf	-2.214**
		TSSMs	-9.475***
	TSSM-fed leaf	TSSMs	-10.21***
9-*cis*-β-carotene	Healthy Leaf	TSSM-fed leaf	-21.22**
		TSSMs	n.a.
	TSSM-fed leaf	TSSMs	n.a
γ-carotene	Healthy Leaf	TSSM-fed leaf	2.541**
		TSSMs	-4.512***
	TSSM-fed leaf	TSSMs	-6.311***
TCrC	Healthy Leaf	TSSM-fed leaf	37.921**
		TSSMs	-640.392***
	TSSM-fed leaf	TSSMs	-678.313***

n.a. "not applicable" due to lack of the compound in the sample; TCrC, total carotenoid content
