# Supplementary material for: Biology of Two-Spotted Spider Mite (Tetranychus urticae): Ultrastructure, Photosynthesis, Guanine Transcriptomics, Carotenoids and Chlorophylls Metabolism, and Decoyinine as a Potential Acaricide
Source: Int J Mol Sci. 2023 Jan 15;24(2):1715. doi: 10.3390/ijms24021715 (PMC9864819; doi:10.3390/ijms24021715)
Supplement: Supplementary file 1 [file ijms-24-01715-s001.zip › Supplementary Table S3.pdf]

Supplementary Table S3. Primers list

Gene ID	Homolog	Forward primer (5' to 3')	Reverse primer (5' to 3')	Product size (bp)
tetur05g00900	*PRPS1*	TTGGTGAAAGTGTCCGTGGT	GCTCCAGCCACAGAAAGCAT	232
tetur01g13720	*PPAT*	TATGGGACAATCGCTGTGGC	GAACTGATCTGGTGGTGGCA	150
tetur05g04480	*GART*	TTTGGTGACGCTGGAAACTC	AAAAGGCACCCATTCCTCCA	172
tetur08g02830	*FGAMS*	TGGGCAAAGGAAAAGAAGCC	ACCCCTGAAGAACCAGTGTC	185
tetur01g13710	*PAICS*	TGGCCTAATGGTGATAAGCGG	CTTCAGGTGGAGGAGGAGGTA	193
tetur02g08320	*ADSL*	AAGGCAGCGCCCATTATTCA	TCGATACAACGAGCCAACCT	119
tetur06g05840	*ATIC*	CTCTGACCAGCGTGACATGA	GCTCCAGCCCGTAAAAGAGT	162
tetur18g02770	*IMPDH1*	CCGAGAGTCCATCATCTGGTT	TGCCAATAGCAGCTCCAACA	201
tetur01g06320	*GMPS*	TGCTCGTTAGTGGTGGTGTC	GGTCGCTTTCGTCTTTACGC	123
tetur21g00550	*PNP*	GCTGGCCATGATGGTGTACT	TTTCGGGTGCATGTCCTTCA	100
tetur01g18640	*Histone H3*	TCACCAATCGTTGGAAAGGT	GGCTGCTCGTAAATCTGCTC	146
