# Supplementary material for: Biology of Two-Spotted Spider Mite (Tetranychus urticae): Ultrastructure, Photosynthesis, Guanine Transcriptomics, Carotenoids and Chlorophylls Metabolism, and Decoyinine as a Potential Acaricide
Source: Int J Mol Sci. 2023 Jan 15;24(2):1715. doi: 10.3390/ijms24021715 (PMC9864819; doi:10.3390/ijms24021715)
Supplement: Supplementary file 1 [file ijms-24-01715-s001.zip › Supplementary Table S4.pdf]

Supplementary Table S4. Chromatographic gradient used for the separation of Chlorophyll, chlorophyll degradation products, and carotenoids.

Time (min)	Solvent A (%)	Solvent B (%)
0	95	5
5	60	40
10	50	50
12	48	52
14	45	55
16	40	60
18	40	60
20	20	80
22	20	80
24	5	95
27	5	95
27.01	95	5
30	95	5
0	95	5
